# Migraine-provoking substances evoke periorbital allodynia in mice

**DOI:** 10.1186/s10194-019-0968-1

**Published:** 2019-02-14

**Authors:** Francesco De Logu, Lorenzo Landini, Malvin N. Janal, Simone Li Puma, Francesco De Cesaris, Pierangelo Geppetti, Romina Nassini

**Affiliations:** 1 Department of Health Sciences, Section of Clinical Pharmacology and Oncology, University of Florence, Viale Pieraccini 6, 50139 Florence, Italy; 2 Department of Epidemiology and Health Promotion, New York University College of Dentistry, New York, USA; 3 Headache Centre, Careggi University Hospital, University of Florence, Florence, Italy

**Keywords:** Migraine, calcitonin gene related peptide, pituitary adenylyl cyclase activating peptide, prostaglandin, histamine, vasoactive intestinal polypeptide, allodynia

## Abstract

**Background:**

Administration of endogenous mediators or exogenous chemicals in migraine patients provoke early headaches and delayed migraine-like attacks. Although migraine provoking substances are normally vasodilators, dilation of arterial vessels does not seem to be the sole contributing factor, and the underlying mechanisms of the delayed migraine pain are mostly unknown. Sustained mechanical allodynia is a common response associated with the local administration of various proalgesic substances in experimental animals and humans. Here, we investigated the ability of a series of endogenous mediators which provoke or do not provoke migraine in patients, to cause or not cause mechanical allodynia upon their injection in the mouse periorbital area.

**Methods:**

Mechanical allodynia was assessed with the von Frey filament assay. Stimuli were given by subcutaneous injection in the periorbital area of C57BL/6J mice; antagonists were administered by local and systemic injections.

**Results:**

Calcitonin gene related peptide (CGRP), but not adrenomedullin and amylin, pituitary adenylyl cyclase activating peptide (PACAP), but not vasoactive intestinal polypeptide (VIP), histamine, prostaglandin E_2_ (PGE_2_) and prostacyclin (PGI_2_), but not PGF_2α,_ evoked a dose-dependent periorbital mechanical allodynia. The painful responses were attenuated by systemic or local (periorbital) administration of antagonists for CGRP (CLR/RAMP1), PACAP (PAC-1), histamine H_1_, PGE_2_ (EP_4_), and PGI_2_ (IP) receptors, respectively.

**Conclusions:**

The correspondence between substances that provoke (CGRP; PACAP, histamine, PGE_2_, PGI_2_), or do not provoke (VIP and PGF_2α_), migraine-like attacks in patients and periorbital allodynia in mice suggests that the study of allodynia in mice may provide information on the proalgesic mechanisms of migraine-provoking agents in humans. Results underline the ability of migraine-provoking substances to initiate mechanical allodynia by acting on peripheral terminals of trigeminal afferents.

## Background

Migraine is a pain disorder that affects about 15% of the adult population worldwide. Thus, the burden of migraine is enormous in terms of suffering, disability, healthcare, and social and economic costs [1]. For these reasons, migraine is ranked among the most disabling medical conditions [2]. Although considerable progress has been made in the development of new treatment options [3, 4], our current understanding of the mechanisms underlying migraine pain is incomplete. Migraine attacks are elicited by a variety of provoking agents [5], and this peculiar feature provides an opportunity to explore disease mechanisms by endogenous mediators or exogenous chemicals that provoke migraine-like attacks in patients [6].

A prototypical example of a migraine-provoking agent is glyceryl trinitrate (GTN). Occupational exposure to, or treatment with, organic nitrates has long been known to provoke headaches [7–10]. Typically, GTN causes an early, mild and short-lived headache minutes after administration, followed by a remarkably delayed migraine-like attack hours later [9, 10]. The ability of GTN to provoke the mild/early headache is temporally associated with the short-lived (<10 min) release of nitric oxide (NO) [11] and ensuing vasodilation [12]. However, the prolonged migraine-like attacks typically begin with a remarkable delay of hours, underlying the temporal dissociation between the early vasomotor response and the delayed proalgesic effect [6, 13, 14]. Thus, the vascular response can hardly explain the delayed migraine-like attack, which, therefore, implicates additional mechanisms. Recently, we reported that GTN administration in mice evokes an early and short-lived (10 minutes) vasodilatation due to a direct vascular action of NO, and a delayed and prolonged (8 hours) periorbital mechanical allodynia (PMA) that is independent from vascular changes and is due to the activation of an oxidative stress-mediated pathway in the soma of trigeminal primary sensory neurons [15]. We also observed that GTN-evoked PMA in mice exhibits a temporal pattern [15] similar to the migraine-like attacks in patients, which are characterized by delayed onset and prolonged duration [6].

In the last three decades, rigorous studies with randomized, double blind and crossover designs have been undertaken, resulting in a systematic investigation of the ability of a series of endogenous mediators or exogenous chemicals to provoke early headaches and delayed migraine-like attacks [6]. Vasodilatation has been proposed as the underlying mechanism of migraine headaches [16]. Notably, both intra and extracranial artery vasodilatation or only intracranial artery vasodilatation have been reported in association with spontaneous migraine attacks [17–19]. Although vasodilatation is elicited by a majority of the migraine provoking agents [6, 14, 20], the vascular response does not seem essential for generating delayed migraine attacks, as robust vasodilators, such as the vasoactive intestinal polypeptide (VIP) or adrenomedullin, do not induce migraine [21, 22]. Thus, an experimental animal model that explores the correspondence between the pain-producing ability of mediators that provoke migraine might be useful for a better understanding of the pro-migraine action of such mediators.

Here, we have investigated whether a series of endogenous mediators, which have been found to provoke or not provoke migraine-like attacks in patients, elicit or do not elicit delayed and prolonged PMA after their injection in the periorbital skin of mice. Provocation tests in humans are usually performed by systemic administration of the stimulus [6]. However, in the present study in mice the local administration was purposively chosen to investigate the interaction between the various mediators and the peripheral terminals of trigeminal nociceptors. These mediators include calcitonin gene-related peptide (CGRP), adrenomedullin, amylin, pituitary adenylyl cyclase activating peptide (PACAP), VIP, histamine, prostaglandin E_2_ (PGE_2_), prostacyclin (PGI_2_) and prostaglandin F_2α_ (PGF_2α_). The receptor type implicated in the PMA evoked by each mediator was also studied. A close correspondence was found between agents that provoke/not provoke delayed migraine in patients and PMA in mice. Thus, the study of PMA in mice may provide information on the proalgesic mechanisms that, in humans, result in the development of migraine-like attacks provoked by endogenous mediators and exogenous chemicals.

## Methods

### Animals

In vivo experiments were carried out according to the European Union (EU) guidelines and Italian legislation (DLgs 26/2014, EU Directive application 2010/63/EU) for animal care (research permit #194/2015-PR). C57BL/6J mice (male, 20-22 g, 6-7 weeks old; Envigo, Milan, Italy) were used. Animals were housed in a temperature (20-24°C)- and relative humidity (45-65%) -controlled *vivarium*, maintained on a 12-hour dark/light cycle (light off from 7.00 PM to 7.00 AM), and with free access to food and water. Animal studies were reported in compliance with the ARRIVE guidelines [23]. The total number of C57BL/6J mice used was 486. Group size of n=6 animals for behavioural experiments were determined using G*Power (v3.1) [24] to detect a minimum difference between paired means of 1.4 standard deviations (or 1.8 standard deviations between groups) in post-hoc tests with type 1 and 2 error rates of 5 and 20%, respectively [15]. Allocation concealment was performed using a randomization procedure (http://www.randomizer.org/). Experiments were done in a quiet, temperature-controlled (20-24°C) room between 9.00 AM and 5.00 PM and were performed by an operator blinded to drug treatment. At the end of each experiment, mice were euthanized with inhaled CO_2_ plus 10-50% O_2_.

### Reagents

CGRP, amylin, PACAP, VIP, PGF_2α_, olcegepant, astemizole, ER819762 and Ro1138452 were from Tocris Bioscience (Bristol, UK); adrenomedullin, PACAP6-38, PGE_2_, PGI_2_ and histamine were from Sigma Aldrich (Milan, Italy); the mouse monoclonal anti-CGRP antibody (clone [4901]) and the inactive immunoglobulin (mouse monoclonal IgG2a) were from Abcam (Cambridge, UK)

### Behavioural experiments

#### Treatment protocols

C57BL/6J mice were injected subcutaneously in the periorbital area (p.orb., 10 μl/site) with CGRP, adrenomedullin, amylin, PACAP, VIP, histamine, PGE_2_, PGI_2_ and PGF_2α_ (0.15, 1.5 and 15 nmol) or their vehicles (0.9% NaCl). The subcutaneous injection was performed unilaterally on the right side of the periorbital area. The mouse was restrained by the double handed manual restraint method [25]. Briefly, the mouse was lifted by the base of the tail and placed on a solid surface with one hand and the tail was pulled back. Then, it was quickly and firmly picked up by the scruff of the neck behind the ears with the thumb and index finger of the other hand. In this way, the mouse face was constrained, and the operator was able to inject the tested compound. Injection was performed as quickly as possible by a single operator, with only minimal animal restraint.

Some mice were pre-treated (30 minutes before the stimuli) with intraperitoneal (i.p., 10 ml/kg) olcegepant (1 μmol/kg corresponding to 0.869 mg/kg), astemizole (4 μmol/kg corresponding to 1.8 mg/kg), ER819762 (60 μmol/kg corresponding to 29.4 mg/kg) or their vehicle (4% dimethyl sulfoxide, DMSO, and 4% tween 80 in 0.9% NaCl) and intravenous (i.v., 1 ml/kg) PACAP6-38 (12 nmol/kg corresponding to 48 μg/kg) and Ro1138452 (30 μmol/kg corresponding to 10.4 mg/kg), or their vehicle (0.9% NaCl). Other mice received locally (p.orb., 10 μl, 30 minutes before the stimuli) olcegepant (4 nmol/site), astemizole (10 nmol/site), ER819762 (10 nmol/site), or their vehicle (4% DMSO and 4% tween 80 in 0.9% NaCl) and PACAP6-38 (240 pmol/site) and Ro1138452 (10 nmol/site), or their vehicle (0.9% NaCl), or a mouse monoclonal anti-CGRP antibody or, as a control, a mouse monoclonal IgG2a (both, 60 pmol/site). In another set of experiments, C57BL/6J mice received intraplantar (i.pl., 20 μl, 30 minutes before the stimuli) injections of olcegepant (4 nmol/site), astemizole (10 nmol/site), ER819762 (10 nmol/site), or their vehicle (4% DMSO and 4% tween 80 in 0.9% NaCl), or PACAP6-38 (240 pmol/site) and Ro1138452 (10 nmol/site), or their vehicle (0.9% NaCl).

#### Acute nociceptive test

Immediately after the p.orb. injections, mice were placed inside a plexiglass chamber and spontaneous nociception was assessed by measuring the time (seconds) that the animal spent face rubbing the injected area with its paws [26] over the next 10 minutes. The p.orb. injection with vehicles produced nociceptive behaviour for a maximum of 3 seconds.

#### Periorbital mechanical allodynia

**Fig. 1:**
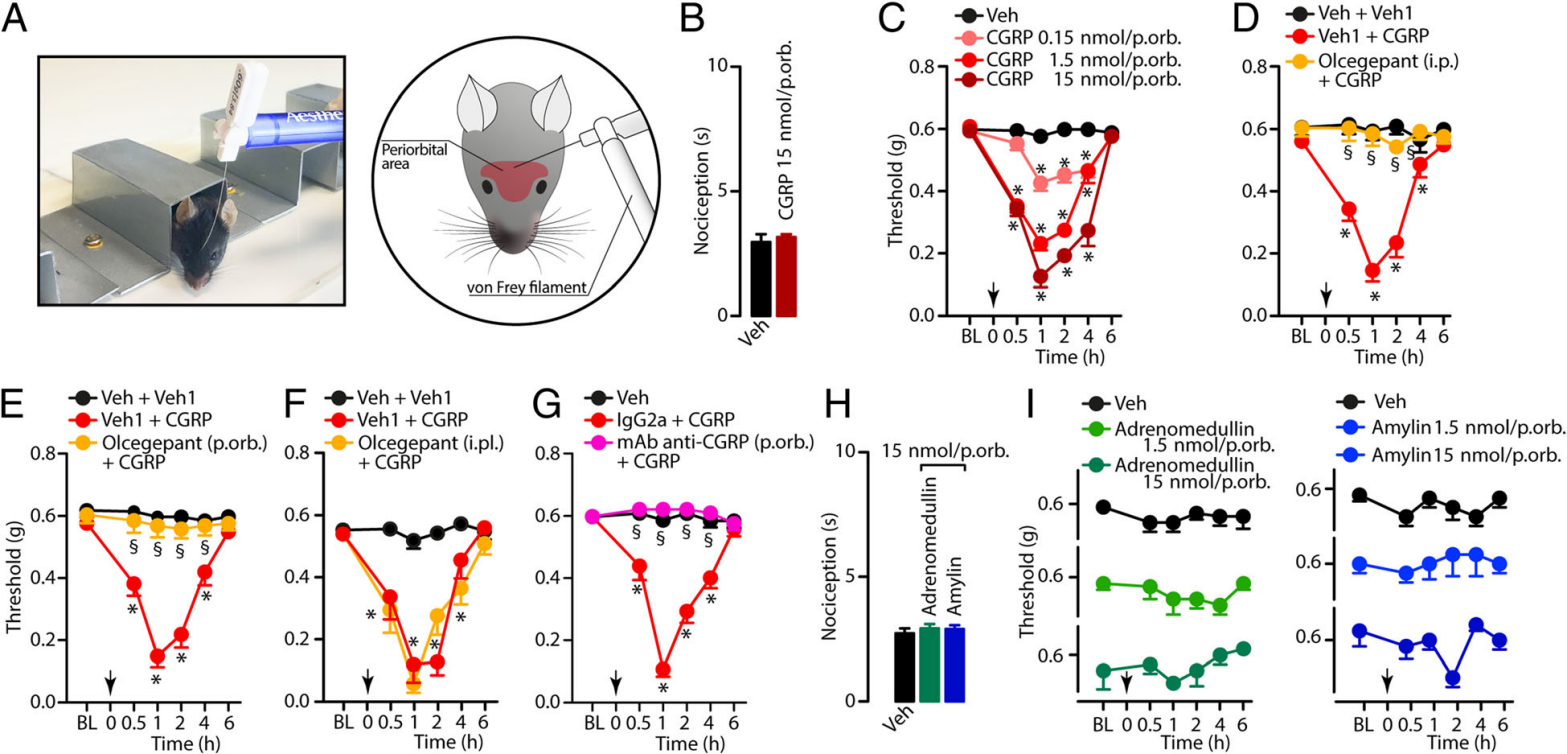
**a** Photograph of restraint apparatus and von Frey monofilament used for evaluating periorbital mechanical allodynia (PMA) and drawing indicating the region where periorbital von Frey stimulus was applied during testing. **b** Subcutaneous injection in the periorbital area (p.orb., 10 μl/15 nmol/site) of CGRP does not cause spontaneous nociceptive behaviour. **c** Dose- and time-dependent PMA evoked by p.orb. Injection of CGRP (10 μl/0.15–15 nmol/site). **d**-**f** Effect of pretreatment with intraperitoneal (i.p. 1 μmol/kg), p.orb. (10 μl/4 nmol/site) and intraplantar (i.pl., 20 μl/4 nmol/site) olcegepant on PMA evoked by CGRP (p.orb., 10 μl/1.5 nmol/site). **g** Effect of pretreatment with a monoclonal anti-CGRP antibody (mAb anti-CGRP) or mouse monoclonal IgG2a (IgG2a) (both p.orb., 10 μl/60 pmol/site) on PMA evoked by CGRP (p.orb., 10 μl/1.5 nmol/site). Adrenomedullin or amylin injection (p.orb., 10 μl/1.5–15 nmol/site) does not evoke spontaneous nociceptive behaviour (**h**) or PMA (**i**). C57BL/6J mice were used. Veh is the vehicle of CGRP (**b**-**g**), adrenomedullin or amylin (**h**, **i**), and veh1 is the vehicle of olcegepant (**d**-**f**). Arrows indicate time of CGRP, adrenomedullin or amylin administration. Olcecepant, mAb anti-CGRP or IgG2a were given 30 min before the stimulus. Error bars indicate mean ± s.e.m., *n* = 6 mice *per* group. *P < 0.05 vs. Veh or Veh + Veh1, ^§^*P <* 0.05 vs. Veh1 + CGRP; two-way ANOVA with Bonferroni post hoc correction

The measurement of PMA was performed by using the up-and-down paradigm as described previously [27, 28] in the same mice in which acute nociceptive responses were monitored for 10 minutes after the stimulus. Animals were allocated in a restraint apparatus designed for the evaluation of periorbital mechanical thresholds. The apparatus consists in an individual clear three-walled plexiglass box (4 H × 4 W x 10 L cm) with an opening for the tail and one for the head and front paws, located on a platform to allow the operator to access to the periorbital area. The box size allowed for head and forepaw movements but prevented the animal from turning around inside it (Fig 1A). One day before the first behavioural observations, mice were habituated to the apparatus. PMA was evaluated in the periorbital region over the rostral portion of the eye (*i.e*., the area of the periorbital region facing the sphenoidal rostrum) of the mice [29] (Fig 1a), before (basal threshold) and after (0.5, 1, 2, 4, 6, 8 hours) treatment.

The day of the experiment, after 20 minutes of adaptation inside the chamber, a series of 7 Von Frey filaments in logarithmic increments of force (0.02, 0.04, 0.07, 0.16, 0.4, 1.0 and 1.4 g) were applied to the periorbital area perpendicular to the skin, with sufficient force to cause slight buckling, and held for approximately 5 seconds to elicit a positive response. The response was considered positive by the following criteria: mouse vigorously stroked its face with the forepaw, head withdrawal from the stimulus, or head shaking. The stimulation initiated with the 0.16 g filament. Absence of response after 5 seconds led to the use of a filament with increased weight, whereas a positive response led to the use of a weaker (*i.e.* lighter) filament. Six measurements were collected for each mouse or until four consecutive positive or negative responses occurred. The 50% mechanical withdrawal threshold (expressed in g) was then calculated from these scores by using a δ value of 0.205, previously determined.

### Statistical Analysis

All data were expressed as mean ± s.e.m. Statistical analysis was performed by the unpaired two-tailed Student’s t-test for comparisons between two groups. Group means for single factor experiments were analysed with a one-way ANOVA, while behavioural experiments with repeated measures employed a two-way mixed model ANOVA, first to determine the presence of an interaction effect, and then to compare the control and treated groups of mice at each time point tested. In both cases, post-hoc comparisons employed the Bonferroni criterion to maintain the experiment-wise error rate at 5%. To avoid uncertainties that would follow from the use of these parametric methods on data that may not attain an interval level of measurement, as well as the potential violation of other ANOVA assumptions, including that of normal sampling distribution, analyses were repeated using non-parametric methods. Both methods led to similar conclusions, and we presented only the parametric analyses, which maintain the original, and more intuitive, units of measure. Statistical analyses were performed on raw data using Prism 5 GraphPad software (GraphPad Software Inc., San Diego, CA, USA), as well as IBM SPSS (v.25, IBM Corp., Armonk, NY, USA). P<0.05 was considered statistically significant.

## Results

### CGRP, adrenomedullin, amylin

CGRP, amylin and adrenomedullin belong to the larger calcitonin family of peptides, which activate, with different potencies, a series of receptors resulting from the multiple combinations of the 3 forms of the calcitonin (CT, further divided into the a, b and δ(1-47)b subtypes) receptor and the CT receptor-like receptor (CLR) with the 3 forms of receptor-activity-modifying proteins (RAMPs) [30]. Although CGRP can bind to all these receptor complexes, it exhibits a higher affinity for the RAMP1/CLR [30]. Adrenomedullin binds with higher potency to the RAMP2-3/CLR and amylin to the RAMP1/CT(a) and the RAMP1-2/CT(b) [30]. Whereas periorbital (p.orb., 10 μl/site) injection of CGRP (0.15, 1.5 and 15 nmol/site), even at the highest dose, did not evoke an acute spontaneous nociceptive response (Fig. 1b), it did cause a robust, dose-dependent and sustained PMA (Fig. 1c). The prolonged PMA was present at 0.5 hour, peaked at 2 hours and declined, to return to baseline values, 6 hours after CGRP injection. Systemic intraperitoneal (i.p., 1 μmol/kg) or local (p.orb., 4 nmol/site), but not intraplantar (i.pl., 20 μl, 4 nmol/site) injection (30 minutes before CGRP) of the CGRP receptor antagonist, olcegepant, prevented PMA (Fig. 1d-f). Furthermore, p.orb. (10 μl) pretreatment (30 min before) with a monoclonal anti-CGRP antibody (60 pmol/site), but not with the inactive mouse monoclonal IgG2a, also prevented the development of PMA induced by p.orb. CGRP (Fig. 1g).

Local (p.orb., 10 μl) administration of adrenomedullin or amylin at the same pro-allodynic dose of CGRP (1.5 or 15 nmol/site), was unable to produce any measurable acute nociceptive response, even at the highest dose. Adrenomedullin or amylin also failed to produce PMA over the entire period of observation (6 hours) (Fig. 1h, i).

### PACAP and VIP

**Fig. 2:**
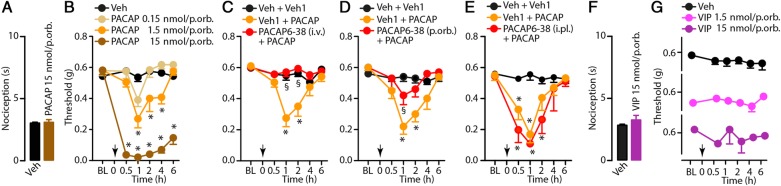
**a** PACAP periorbital (p.orb., 10 μl/15 nmol/site) injection does not evoke spontaneous nociceptive behaviour. **b** Dose- and time-dependent periorbital mechanical allodynia (PMA) evoked by p.orb. Injection of PACAP (10 μl/0.15–15 nmol/site). **c**-**e** Effect of pretreatment with intravenous (i.v., 12 nmol/kg), p.orb. (10 μl/240 pmol/site) or intraplantar (i.pl., 20 μl/240 pmol/site pmol) PACAP6–38 on PMA evoked by PACAP (p.orb., 10 μl/1.5 nmol/site). VIP injection (p.orb., 10 μl/1.5 and 15 nmol/site) does not evoke spontaneous nociceptive behaviour (**f**) or PMA (**g**). C57BL/6J mice were used. Veh is the vehicle of PACAP (**a**-**e**) or VIP (**f**, **g**) and veh1 is the vehicle of PACAP6–38 (**c**-**e**). Arrows indicate time of PACAP or VIP administration. PACAP6–38 was given 30 min before the stimulus. Error bars indicate mean ± s.e.m., *n* = 6 mice *per* group. *P < 0.05 vs. Veh or Veh + Veh1, ^§^*P <* 0.05 vs. Veh1 + PACAP; two-way ANOVA with Bonferroni post hoc correction

The members of the family of the PACAP and VIP vasoactive peptides act on VPAC-1 and VPAC-2 receptors with comparable affinity, whereas the PAC-1 receptor isoform has 100-fold higher affinity for PACAP [31, 32]. Local (p.orb., 10 μl) injection of PACAP (0.15, 1.5 and 15 nmol/site), which did not provoke any detectable spontaneous nociceptive behaviour even at the highest dose, induced a marked, dose-dependent and sustained (1-6 hours) PMA (Fig. 2a, b). Intravenous (i.v., 1 ml/kg, 12 nmol/kg) or p.orb. (10 μl, 240 pmol/site), but not i.pl. (20 μl, 240 pmol/site), pretreatment with the selective PACAP receptor antagonist, PACAP6-38, prevented PACAP-induced PMA (Fig. 2c, e). VIP (1.5 or 15 nmol/site, p.orb.) was unable to produce either acute nociception or PMA (Fig. 2f, g).

### Histamine

**Fig. 3:**
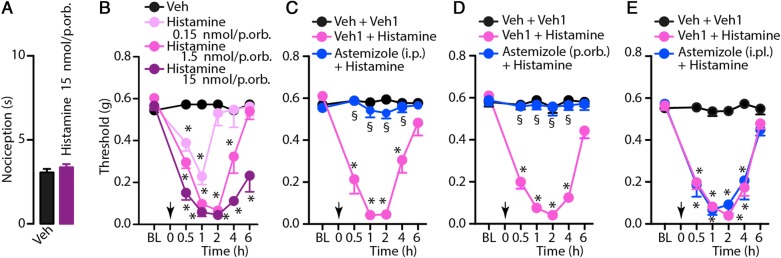
**a** Histamine periorbital (p.orb., 10 μl/15 nmol/site) injection does not evoke spontaneous nociceptive behaviour. **b** Dose- and time-dependent periorbital mechanical allodynia (PMA) evoked by p.orb. Injection of histamine (10 μl/0.15–15 nmol/site). **c**-**e** Effect of pretreatment with intraperitoneal (i.p., 4 μmol/kg), p.orb. (10 μl/10 nmol/site) or intraplantar (i.pl., 20 μl/10 nmol/site) astemizole on PMA evoked by histamine (p.orb., 10 μl/1.5 nmol/site). C57BL/6J mice were used. Veh is the vehicle of histamine and veh1 is the vehicle of astemizole (**c**-**e**). Arrows indicate time of histamine administration. Astemizole was given 30 min before histamine. Error bars indicate mean ± s.e.m., *n* = 6 mice *per* group. *P < 0.05 vs. Veh or Veh + Veh1, ^§^*P <* 0.05 vs. Veh1 + histamine; two-way ANOVA with Bonferroni post hoc correction

Histamine is a ubiquitous mediator released from mast cells, enterochromaffin-like cells and neurons, implicated in pathophysiological responses such as arousal state, allergy, inflammation, itch and pain [33–35]. Its actions are mediated by four distinct receptors, the H_1_, H_2_, H_3_ and H_4_ receptors [36]. Local injection (p.orb., 10μl) of histamine (0.15, 1.5 and 15 nmol/site) was unable to produce any spontaneous acute nociception, even at the highest dose administered, but induced a dose-dependent and sustained (4-6 hours) PMA (Fig. 3a, b). Systemic (i.p., 10 ml/kg, 4 μmol/kg) or p.orb. (10 μl, 10 nmol/site), but not i.pl. (20 μl, 10 nmol/site) pretreatment with the histamine H_1_ receptor antagonist, astemizole, prevented histamine-induced PMA (Fig. 3c-e).

### PGE_2_, PGI_2_, PGF_2α_

Prostanoids are ubiquitous mediators which play a major role in a large variety of physiological responses and pathological process, including inflammation and pain [37]. Cyclooxygenase inhibition by non-steroidal anti-inflammatory drugs (NSAIDs), which prevents the transformation of arachidonic acid into the inactive prostaglandin precursor, prostaglandin H_2_ (PGH_2_), is a mainstay of the acute migraine attack, thus implicating prostaglandins in migraine pain [38, 39]. PGE_2_ administration in the mouse paw is known to evoke spontaneous acute nociception [40]. Accordingly, we found that PGE_2_ (0.15, 1.5 and 15 nmol/site), but not PGI_2_ (0.15, 1.5 and 15 nmol/site) or PGF_2α_ (1.5-15 nmol/site) injection into the mouse periorbital skin elicited a marked spontaneous nociceptive response (

**Fig. 4:**
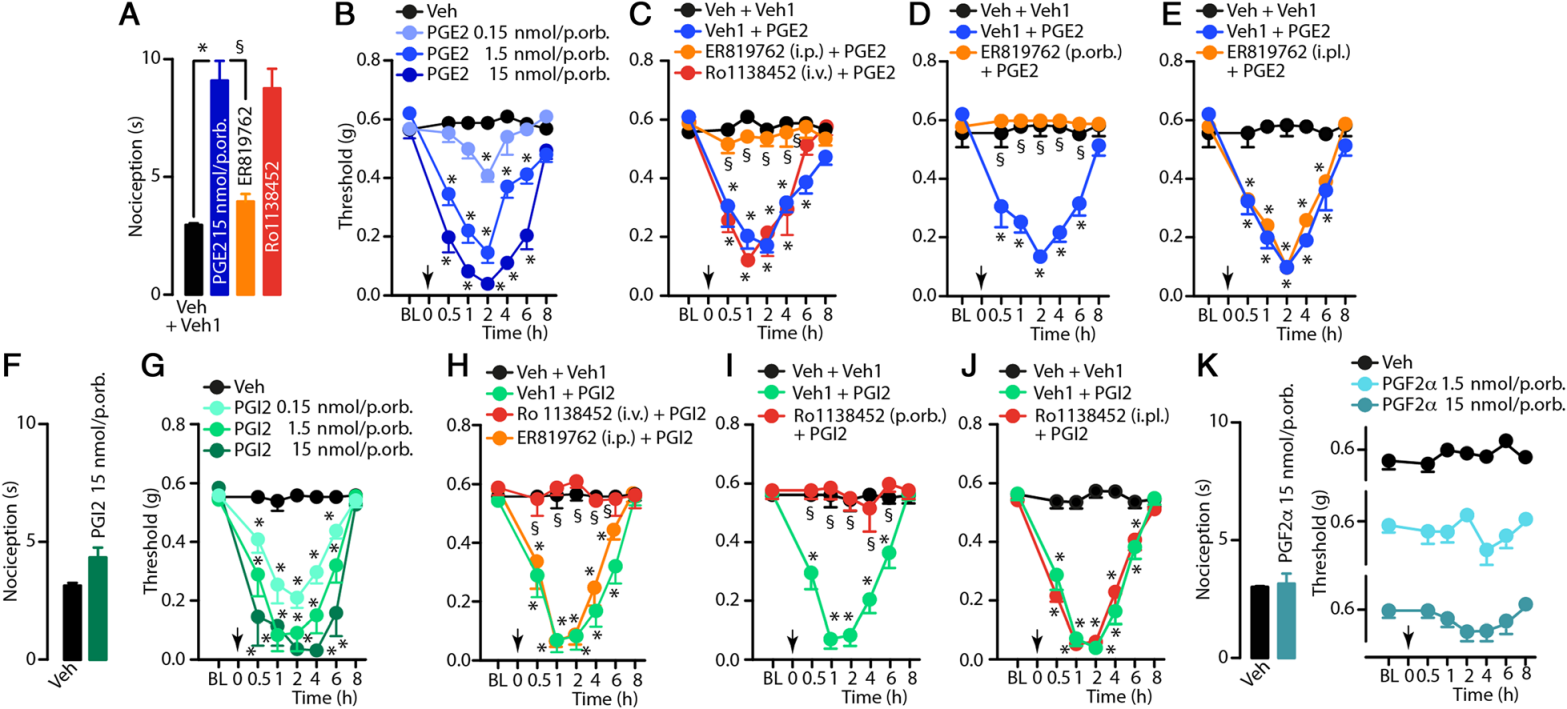
**a** Effect of pretreatment with intraperitoneal (i.p., 60 μmol/kg) ER819762 or intravenous (i.v., 30 μmol/kg) Ro1138452 on the spontaneous nociceptive behaviour evoked by periorbital (p.orb., 10 μl/15 nmol/site) injection of PGE_2_. **b** Dose- and time-dependent periorbital mechanical allodynia (PMA) evoked by p.orb. Injection of PGE_2_ (10 μl/0.15–15 nmol/site). **c** Effect of pretreatment with ER819762 (i.p., 60 μmol/kg) and Ro1138452 (i.v., 30 μmol/kg) on PMA evoked by PGE_2_ (p.orb., 10 μl/1.5 nmol/site). **d**, **e** Effect of pretreatment with p.orb. (10 μl/10 nmol/site) or intraplantar (i.pl., 20 μl/10 nmol/site) ER819762 on PMA evoked by PGE_2_ (p.orb., 10 μl/1.5 nmol/site). **f** PGI_2_ (p.orb., 10 μl/15 nmol/site) does not evoke spontaneous nociceptive behaviour. **g** Dose- and time-dependent PMA evoked by PGI_2_ (p.orb., 10 μl/0.15–15 nmol/site). **h** Effect of pretreatment with Ro1138452 (i.v., 30 μmol/kg), and ER819762 (i.p., 60 μmol/kg) on PMA evoked by PGI_2_ (p.orb., 10 μl/1.5 nmol/site). **i**, **j** Effect of pretreatment with p.orb. (10 μl/10 nmol/site) or i.pl. (20 μl/10 nmol/site) Ro1138452 on PMA evoked by PGI_2_ (p.orb., 10 μl/1.5 nmol/site). **k** PGF_2α_ (p.orb., 10 μl/1.5–15 nmol/site) does not evoke spontaneous nociceptive behaviour or PMA. C57BL/6J mice were used. Veh is the vehicle of PGE_2_ (**a**-**e**), PGI_2_ (**f**-**j**) or PGF_2α_ (**k**) and veh1 is the vehicle of ER819762 (**d**, **e**), Ro1138452 (**i, j**) or a combination of vehicles of ER819762 and Ro1138452 (**a**, **c**, **h**). Arrows indicate time of PGE_2_, PGI_2_ or PGF_2α_ administration. ER819762 and Ro1138452 were given 30 min before PGE_2_ or PGI_2_. Error bars indicate mean ± s.e.m., *n* = 6 mice *per* group. *P < 0.05 vs. Veh or Veh + Veh1, ^§^*P <* 0.05 vs. Veh1 + PGE_2_ or Veh1 + PGI_2_; one- or two-way ANOVA with Bonferroni post hoc correction

Fig. 4a, f, k). Furthermore, injection of both PGE_2_ and PGI_2_, but not PGF_2α_, evoked a dose-dependent sustained (0.5-6 hours) PMA (Fig. 4b, g, k). Pretreatment with i.p. (10 ml/kg, 60 μmol/kg) and p.orb. (10 μl, 10 nmol/site), but not i.pl. (20 μl, 10 nmol/site) prostaglandin receptor 4 (EP_4_) antagonist, ER819762, prevented PGE_2_-induced spontaneous nociception and PMA (Fig. 4a, c-e). Pretreatment with i.v. (1 ml/kg, 30 μmol/kg) and p.orb., (10 μl, 10 nmol/site), but not i.pl. (20 μl, 10 nmol/site) antagonist for the prostacyclin receptor (IP), Ro1138452, prevented PGI_2_-induced PMA (Fig. 4h-j). Conversely, Ro1138452 (i.v., 30 μmol/kg) did not affect spontaneous nociception and PMA evoked by PGE_2_ and ER819762 (i.p., 60 μmol/kg) did not affect PMA evoked by PGI_2_ (Fig. 4a, c, h).

## Discussion

The members of the calcitonin family of peptides activate a variety of receptors deriving from the dimerization of CLR or CL with RAMP proteins. Adrenomedullin, which stimulates the combinations of the CLR with RAMP2 or RAMP3 with a potency higher than CGRP (AM_1_ and AM_2_ receptor, respectively), and amylin, which is equipotent to CGRP on the receptor combinations formed by the three CT subtypes with RAMP1, RAMP2 or RAMP3, failed to evoke allodynia. A possible effect of amylin and adrenomedullin on the RAMP1/CLR, or of CGRP on the different combinations of receptors for amylin and adrenomedullin has been claimed to contribute to the pro-migraine action of CGRP or its receptor [30]. As previously reported in the mouse hindpaw [41] and periorbital area [15], we confirm that CGRP causes a robust and sustained mechanical allodynia, which is attenuated by both systemic and local administration of the selective RAMP1/CLR (CGRP receptor) antagonist, olcegepant. The observation that, under the same experimental conditions neither adrenomedullin nor amylin evoked PMA negates the implication of their preferred receptors in CGRP-mediated pain-like responses. Furthermore, the present results do not support the hypothesis that amylin or adrenomedullin act on RAMP1/CLR to evoke pain-like responses. Previous results that CGRP administration to migraineurs induced delayed migraine-like attacks [42], whereas adrenomedullin was found to be inactive [22], strengthened and excluded the role in migraine mechanism of CGRP and adrenomedullin, respectively. The present findings on the calcitonin related peptides, recapitulating human results, support the predictive value of mouse PMA in investigating pain mechanisms of migraine.

Clinical trials with anti CGRP or anti RAMP1/CLR monoclonal antibodies, while showing excellent efficacy and safety profile, also indicate that a subset of migraine patients either do not respond or have a partial benefit [4, 43, 44]. This observation suggests that additional mechanisms and mediators contribute to migraine pain, thus prompting the study of substances other than CGRP. PGE_2_ and PGI_2_, two prostaglandins that induce headaches and migraine-like attacks in humans [45–48], elicited PMA in mice. In contrast, PGF_2α_, a prostaglandin, which was unable to evoke migraine-like attack in patients [49], failed to elicit PMA in mice. The use of selective antagonists for the EP_4_ and IP receptors showed that PGE_2_ and PGI_2_ caused allodynia exclusively by activating the respective preferred receptor. This conclusion suggests that in humans PGE_2_ and PGI_2_ elicit migraine-like attacks by acting on EP_4_ and IP receptors, respectively. As reported previously, in the mouse hindpaw [40], PGE_2_ was the sole compound among all the presently investigated substances that evoked an early spontaneous nociceptive response, which, similarly to allodynia, was abated by EP4 receptor antagonism. However, given that only one of the migraine-provoking substances elicited spontaneous nociception, the significance of such early non-evoked pain-like responses for migraine pain mechanism remains unclear.

Histamine, a key proinflammatory and allergic mediator with a proalgesic role provokes migraine-like attacks in patients [50–52]. Furthermore, anecdotical reports and clinical investigations [53] have proposed the use of increasing doses of histamine to desensitize the pain-producing mechanism in migraine patients. Present data show that, by targeting the H_1_ receptor subtype, histamine evokes PMA in mice and provides indirect support to the contribution of the H_1_ receptor, rather than H_2_ receptors [51], in provoking migraine [52], and to the desensitization process that is supposed to ameliorate migraine [53]. It should be underlined that, despite the ability of histamine to sensitise nociceptors *via* H_1_ receptor activation, the H_1_-antagonists were not always effective in reducing migraine [54].

VIP and PACAP, which belong to the glucagon/secretin family of regulatory peptides, stimulate three distinct receptors: the PAC-1, selectively activated by PACAP, and the VPAC-1 and VPCA-2, which are equipotently activated by both PACAP and VIP. The observation that PACAP, but not VIP, elicited allodynia, suggests that the PACAP/PAC-1 is the sole pathway implicated in generating pain-like responses. PACAP and VIP are both vasodilator substances [55, 56]. The ability of PACAP, and not VIP, to cause allodynia in mice and migraine-like attacks in humans [21, 57], supports the hypothesis that vasodilatation is not *per se* a major factor contributing to allodynia in mice and migraine pain in humans. These findings are in line with previous observations that PACAP, but not VIP, causes delayed activation and sensitization of central trigeminovascular neurons *via* activation of the PAC1 receptor [58]. The implication of mast cells has been proposed in the pathway activated by PACAP to elicit pain. Mast cells may release PACAP [59], and PACAP, *via* a hitherto uncharacterized receptor, degranulates mast cells [60]. The present model could be used to further explore local mechanisms that, activated by PACAP and implicating mast cells, result in pain responses.

The underlying mechanism that promotes migraine attacks is unclear. Clinical investigation with small molecule CGRP receptor antagonists underscores the key role of CGRP in the genesis of migraine pain [4, 61]. However, the specific site(s) of the proalgesic action of CGRP in migraine pain remains elusive. Recent clinical trials with monoclonal antibodies that block CGRP or its receptor [43, 44], underline the hypothesis that the pain produced by CGRP during migraine attacks originates at a peripheral site, outside the blood brain barrier. However, the precise location of such a peripheral site is uncertain. The observation that PMA was attenuated only if antagonists were given locally, close to (p.orb.), but not at distance from (i.pl.) the site where the respective agonists were injected, indicates that the anatomical site where pain hypersensitivity initially originates is the terminal region of peripheral trigeminal fibres.

Differences may exist between the trigeminal fibres of the skin and those innervating meningeal blood vessels [62, 63] that are possibly implicated in migraine pathogenesis. Nevertheless, the local subcutaneous injection of stimuli was adopted purposively to selectively investigate the interaction between pro-migraine mediators and peripheral terminals of trigeminal nociceptors and to minimize confounding factors, deriving from the systemic administration or the surgical procedures required for dural application of the stimuli. The old dispute regarding the contribution of the peripheral or central nervous system to allodynia and hyperalgesia [64–67] has not yet been completely resolved. The present investigation reports a condition of hypersensitivity that originates peripherally in the periorbital area of mice, but by no means implies that central neural pathways do not contribute to sustain allodynia. However, pathways and mechanisms regulating mechanical hypersensitivity in the central nervous system are not the object of the present study. Clinical investigation shows that, while blockade of the CGRP system provides benefit in a large proportion of patients, a subset of migraineurs appears to be resistant [43, 44], thus suggesting that additional mediators and mechanisms contribute to migraine pain. The ability to evoke PMA in mice adds support to the role of additional migraine provoking mediators in spontaneous pain attacks.

## Conclusions

The major finding of the present study is the strict correspondence between mediators that provoke migraine in patients and evoke periorbital allodynia in mice. The same correspondence was observed between mediators that do not provoke migraine in patients and do not evoke allodynia in mice. An additional relevant finding is that, although most of the pro-allodynic substances tested in the present study are vasodilators, two robust vasodilators, VIP and adrenomedullin, did not evoke allodynia, thus indicating that vascular activity is not *per se* sufficient to elicit pain. Cutaneous allodynia is frequently reported by migraine patients during the attack [68, 69]. However, it should be considered that migraine-like attacks induced by provoking substances are characterized by delayed and prolonged spontaneous, non-evoked pain. Therefore, mechanical allodynia cannot recapitulate the complete spectrum of the pain modalities experienced by migraineurs during their attacks. Nevertheless, disclosing the mechanisms used by the different mediators, and particularly CGRP, to evoke delayed and sustained mechanical allodynia in mice may provide insights for a better understanding of the mechanisms by which the same substances generate migraine pain in patients.

## Abbreviations

ANOVA: Analysis of variance
CGRP: Calcitonin gene related peptide
CLR: CT receptor-like receptor
CT: Calcitonin
DMSO: Dimethyl sulfoxide
EP_4_: Prostaglandin receptor 4
GTN: Glyceryl trinitrate
i.p.: Intraperitoneal
i.pl.: Intraplantar
i.v.: Intravenous
IP: Prostacyclin receptor
NSAIDs: Non-steroidal anti-inflammatory drugs
p.orb.: Periorbital
PACAP: Pituitary adenylyl cyclase activating peptide
PG: Prostaglandin
PGI_2_: Prostacyclin
PMA: Periorbital mechanical allodynia
RAMPs: Receptor-activity-modifying proteins
VIP: Vasoactive intestinal polypeptide
VPAC: VIP receptor
